# Development and validation of a new drug-focused predictive risk score for postoperative delirium in orthopaedic and trauma surgery patients

**DOI:** 10.1186/s12877-024-05005-1

**Published:** 2024-05-13

**Authors:** Carolin Geßele, Thomas Saller, Vera Smolka, Konstantinos Dimitriadis, Ute Amann, Dorothea Strobach

**Affiliations:** 1 Hospital Pharmacy, LMU University Hospital, LMU Munich, Munich, Germany; 2 Doctoral Program Clinical Pharmacy, LMU University Hospital, LMU Munich, Munich, Germany; 3 Department of Anaesthesiology, LMU University Hospital, LMU Munich, Munich, Germany; 4 Department of Orthopaedics and Trauma Surgery, LMU University Hospital, LMU Munich, Munich, Germany; 5 Department of Neurology, LMU University Hospital, LMU Munich, Munich, Germany; 6 Faculty of Medicine, LMU Munich, Munich, Germany

**Keywords:** Medication Safety, Geriatrics, Screening tools, Postoperative delirium

## Abstract

**Background:**

Postoperative delirium (POD) is the most common complication following surgery in elderly patients. During pharmacist-led medication reconciliation (PhMR), a predictive risk score considering delirium risk-increasing drugs and other available risk factors could help to identify risk patients.

**Methods:**

Orthopaedic and trauma surgery patients aged ≥ 18 years with PhMR were included in a retrospective observational single-centre study 03/2022-10/2022. The study cohort was randomly split into a development and a validation cohort (6:4 ratio). POD was assessed through the 4 A’s test (4AT), delirium diagnosis, and chart review. Potential risk factors available at PhMR were tested via univariable analysis. Significant variables were added to a multivariable logistic regression model. Based on the regression coefficients, a risk score for POD including delirium risk-increasing drugs (DRD score) was established.

**Results:**

POD occurred in 42/328 (12.8%) and 30/218 (13.8%) patients in the development and validation cohorts, respectively. Of the seven evaluated risk factors, four were ultimately tested in a multivariable logistic regression model. The final DRD score included age (66–75 years, 2 points; > 75 years, 3 points), renal impairment (eGFR < 60 ml/min/1.73m^2^, 1 point), anticholinergic burden (ACB-score ≥ 3, 1 point), and delirium risk-increasing drugs (*n* ≥ 2; 2 points). Patients with ≥ 4 points were classified as having a high risk for POD. The areas under the receiver operating characteristic curve of the risk score model were 0.89 and 0.81 for the development and the validation cohorts, respectively.

**Conclusion:**

The DRD score is a predictive risk score assessable during PhMR and can identify patients at risk for POD. Specific preventive measures concerning drug therapy safety and non-pharmacological actions should be implemented for identified risk patients.

**Supplementary Information:**

The online version contains supplementary material available at 10.1186/s12877-024-05005-1.

## Introduction

Delirium is defined as an acute change in attention, awareness, and cognition [1]. It usually develops rapidly with a fluctuating course [1]. In elderly, hospitalized patients, it represents a severe complication with a prevalence ranging from 20% in general surgery up to 70% in intensive care units [2, 3]. After surgical intervention, postoperative delirium (POD) is one of the most common complications [4]. The clinical consequences of delirium are severe with an increase in mortality, length of hospital stay, and development of dementia or cognitive decline [5, 6].

Multiple risk factors determine the risk for delirium. Predisposing risk factors include advanced age, visual and hearing impairment, history of prior delirium, cognitive impairment, frailty, and comorbidities (i.e. cardiovascular or renal diseases). In addition, precipitating factors such as acute medical illness (i.e. infections, hypoglycaemia), trauma, surgical procedures, dehydration, pain, medication use, and drug withdrawal are relevant [2]. Preventive measures can reduce the occurrence of delirium; therefore, overall non-pharmacological measures are recommended for vulnerable patients [2, 7]. However, identifying patients at risk who are most likely to benefit from specific preventive measures remains challenging. One approach is the use of risk prediction scores based on pre- and perioperative risk factors for the identification of patients at high risk for delirium.

Drugs, especially substances targeting the central nervous system, are a proven risk factor for in-hospital delirium [8–11]. However, a recent systematic review found that medication is not adequately considered in previously developed risk scores and should be addressed in future models [12]. Existing risk scores already considering medication include the risk factors polymedication (≥ five regular drugs) [13], psychoactive and anticholinergic drugs [14], or medication for insomnia treatment [15]. The Delirium Model (DEMO) includes drugs associated with delirium in a linear prediction model [16]. Recently, a medication-based prediction score for POD in surgical patients was developed by our group [17]; however, further revision concerning the weighing of risk factors and testing of the predictive performance is needed.

Importantly, patients at risk for POD should be identified at an early stage prior to surgery. One opportunity for a timely identification of patients at risk for drug-related POD is during pharmacist-led medication reconciliation (PhMR) at hospital admission. In addition, scores including delirium risk-increasing drugs should be easy to use for pharmacists involved in the hospital medication process, and variables should be readily accessible in clinical practice. Consequently, pharmacists can inform physicians about the patients’ individual risk and make preventive suggestions with a focus on minimizing the risk for drug-related POD.

Therefore, the aim of our study was to further develop and validate a risk score for POD including delirium risk-increasing drugs, which can be performed during PhMR at hospital admission by pharmacists based on the admission medication and other available risk factors. This risk score could identify patients at risk for drug-related POD benefiting from suggestions for drug therapy safety and additional preventive measures.

## Methods

### Study design

A retrospective single-centre cohort study was conducted at LMU University Hospital Munich, a tertiary care hospital, from March to October 2022. Ethics approval was obtained from the Ethics Committee of LMU University Hospital Munich (No. 23–0041). Three orthopedic or trauma surgery wards were included, which were part of a pilot project focused on reducing postoperative complications in elderly patients, specifically delirium (gertrud program - age-appropriate proactive health care) [18]. Ward staff (physicians, nurses, and physiotherapists) was especially trained for delirium awareness, and trained nurses regularly performed delirium assessments using the 4 A’s test (4AT) [19]. For patients with a 4AT score ≥ 4, physicians confirmed the result, and if delirium was present, a diagnosis was documented according to the International Classification of Diseases 10th revision (ICD-10) [20].

The inclusion criteria for our study were age ≥ 18 years, surgical procedure in orthopaedics or trauma surgery, and a pharmacist-led medication reconciliation (PhMR) at hospital admission. Patients with preoperative delirium, delirium due to alcohol withdrawal, or cases with missing data were excluded from the analysis.

PhMR at LMU University Hospital is routinely performed for all admitted surgical patients from Monday to Friday to assess a detailed drug history and generate a medication list with prescribed and over-the-counter drugs. In addition, smoking status and alcohol use are assessed according to self-reports. Information is saved in the electronic medication record Meona® (Mesalvo GmbH Freiburg, Germany).

### Identification of potential preoperative risk factors

The previously established medication-based prediction score for POD in surgical patients developed by our group included age (≥ 65 years; ≥ 75 years), male sex, renal impairment (estimated glomerular filtration rate (eGFR) < 60 ml/min/1.73 m^2^), hepatic impairment (model of endstage liver disease (MELD) score 10–14; ≥ 15 [21]), delirium risk-increasing drugs (antidiabetics, opioids, antiepileptic drugs, anti-Parkinson drugs, antipsychotic drugs, hypnotics and sedatives including benzodiazepines, antidepressant drugs, anti-dementia drugs, and antihistamines for systemic use), and anticholinergic burden (ACB score ≥ 3 [22]) [17]. The ACB score is an established score summing up the anticholinergic properties of a patient’s medication; drugs are assigned no (0), weak (1), moderate (2), or strong (3) anticholinergic effects. A literature search was performed on additional risk factors for delirium and risk factors included in other published prediction scores. The identified risk factors were evaluated for availability at the time of PhMR, and a consensus for inclusion in the prediction score was reached following interprofessional discussion by neurologists, geriatricians, anaesthesiologists, and pharmacists.

### Data collection

All patient information, admission medication, laboratory data (eGFR calculated by the CKD-EPI equation [ml/min/1.73 m^2^] [23]), alcohol use, and smoking status were collected from electronic health records (i.s.h.med®, Cerner Corporation, North Kansas City, USA) and Meona® as assessed during PhMR. Alcohol use was classified according to the National Institute on Alcohol Abuse and Alcoholism (NIAAA) [24]. Smoking status was documented as ‘yes’ or ‘no’ regardless of the units consumed per day (cigarettes, cigars, vaporizers). The anticholinergic burden was calculated with the ACB score for drugs available in Germany [22]. Data was documented using Microsoft Excel® 2016 (Seattle, WA, USA).

### Retrospective assessment of delirium diagnoses

POD was assessed for all study patients based on the documented 4AT scores, ICD-10 diagnoses (F05.0, F05.1, F05.8, F05.9 [20]), and a subsequent chart review. A physician confirmed the initial assessment by a pharmacist. In addition, a chart review, as validated in previous studies, was conducted [25, 26] (keywords: delirious, confusion, disoriented, disturbed attention, hallucination, restless, and agitated [16, 27]).

### Statistical analysis

A study size of 550 patients was calculated for ten outcome events per variable [28], seven risk factors and an estimated overall POD prevalence of 12% [29]. Statistical analysis was performed with SPSS Statistics® version 29.0 (IBM Corp., Armonk, NY, USA). For descriptive statistics, categorical variables were expressed in absolute and relative frequencies and compared using Chi^2^-Test or Fisher’s exact test. Continuous variables were expressed as mean ± standard deviation (SD) or as median with interquartile range (IQR). Comparisons were made by Student’s t-test or Mann-Whitney U test as appropriate. *P* values were two-sided, and values < 0.05 were considered statistically significant. Figures were created using Adobe Illustrator® version 27.0 (San Jose, CA, USA).

### Score development and validation

For the development and internal validation of the predictive score for drug-related POD, the cohort was divided into two cohorts by random allocation (split-sample validation approach, 6:4 allocation), and patients were randomly assigned to either cohort through computerized random numbers using Microsoft Excel® 2016 (Seattle, WS, USA).

For the development cohort, univariable logistic regression analysis was used to evaluate the associations between continuous or categorical variables and the presence or absence of POD. Continuous variables were transformed into categorical variables by using suitable cut-off values determined through clinically established definitions for chronic kidney disease [23], geriatric age > 65 years [30] and high anticholinergic burden with an ACB score ≥ 3 [22]. If appropriate, the Youden index of receiver operating characteristic (ROC) analysis was also used.

Statistically significant variables (*p* < 0.05) from univariable logistic regression analysis were added to a multivariable forward stepwise logistic regression model. For derivation of the score, the weighting point for each variable was defined by the corresponding regression coefficient rounded to the nearest integer. The area under the curve (AUC) was obtained through ROC analysis. Optimal cut-off values were determined through the Youden index. Goodness of fit was assessed using the Hosmer-Lemeshow test (*p* > 0.05, good fit), and multicollinearity of variables was reviewed through a correlation matrix.

For validation, the derived score was applied to the patients in the validation cohort, and the corresponding AUC was determined. The sensitivity, specificity, positive predictive value (PPV), negative predictive value (NPV), positive likelihood ratio (LR+), and negative likelihood ratio (LR-) were calculated. The calibration of the model was assessed by plotting a function of the predicted risks against the observed risks.

## Results

### Definition of potential preoperative risk factors

Based on the previously developed medication-based prediction score [17], a renewed literature review, and interprofessional discussions, seven potential risk factors for drug-related POD were established (Table 1). Additional potential risk factors included for further analysis were heavy alcohol use [13, 31–33], daily smoking [14, 25, 31], and inhalants for chronic obstructive airway disease [34]. Due to the large number of missing laboratory values, the MELD score [21] was excluded.

**Table 1:** Definition of potential risk factors for POD available at pharmacist led medication reconciliation [13, 14, 17, 25, 31–34]

Potential risk factor		Comment
Age [years]		
Sex [male/female]		
Kidney function (eGFR) [ml/min/1.73m^2^]		
Delirium risk-increasing drugs [n]	ATC code	Number of drugs for regular and on demand medication
Anti-dementia drugs	N06D	
Antidepressants	N06A	Also for treatment of neuropathic pain
Antiepileptic drugs	N03	Also for treatment of neuropathic pain
Antipsychotics	N05A	
Anti-Parkinson drugs	N04	Also for treatment of restless legs syndrome
Anxiolytics (benzodiazepines)	N05BA	
Hypnotics and sedatives	N05C	
Opioids	N02A	
Antihistamines for systemic use	R06	
Antidiabetics	A10	Oral antidiabetics/GLP-1 analogues summed up as 1 drug; insulins and analogues summed up as 1 drug
Inhalants for chronic obstructive airway disease (COPD)	R03AL, R03BB	Adrenergic + LAMA (+ ICS), LAMA
Anticholinergic burden [ACB score] [22]		
Heavy alcohol use [24] [yes/no]		men (> 14 standard drinks per week/> 4 drinks any day); women (> 7 standard drinks per week/> 3 drinks any day)
Smoking status [yes/no]		Daily smoking of cigarettes (based on self-report)

POD = postoperative delirium; eGFR = estimated glomerular filtration rate; ATC code = Anatomical Therapeutic Chemical code; GLP-1 = Glucagon-like Peptide 1; LAMA = long-acting muscarinic receptor antagonist; ICS = inhaled corticosteroid

### Characterization of the retrospective patient cohort

During the study period, 804 patients were initially screened for inclusion. The inclusion criteria were not met by 218 patients (missing PhMR, *n* = 56; no surgical intervention in orthopaedics or trauma surgery, *n* = 162). A total of 40 patients were excluded due to missing laboratory values (*n* = 38), preoperative delirium (*n* = 1), or delirium due to alcohol withdrawal (*n* = 1). Overall, 546 patients (median age 74 years (IQR 64–82), 45.2% male) were included and randomly divided into development (60%, *n* = 328) and validation (40%, *n* = 218) cohorts. Table 2 shows the patient characteristics, prevalence of POD, and potential risk factors associated with delirium for both study cohorts. A full overview of the observed drug classes of delirium risk-increasing drugs can be found in Supplementary Table S1.

**Table 2:** Patient characteristics and potential risk factors associated with POD in the development and validation cohorts of patients with or without POD

Variable	Development cohort (*n* = 328)			Validation cohort (*n* = 218)		
POD	no	yes	*p*	no	yes	*p*
	286 (87.2)	42 (12.8)		188 (86.2)	30 (13.8)	
Age [years]	72 (61–81)	84 (76–90)	< 0.001^a^	73 (62–81)	87 (81–89)	< 0.001^a^
Sex						
female	153 (53.5)	25 (59.5)	0.464^b^	105 (55.9)	16 (53.3)	0.797^b^
male	133 (46.5)	17 (40.5)		83 (44.1)	14 (46.7)	
eGFR [ml/min/1.73m^2^]	80 (65–93)	55 (38–80)	< 0.001^a^	81 (66–91)	60 (35–82)	< 0.001^a^
ACB score	0 (0–1)	2 (0–3)	< 0.001^a^	0 (0–1)	1 (0–3)	0.001^a^
ACB score ≥ 3 [n]	20 (7.0)	12 (28.6)	< 0.001^b^	15 (8.0)	8 (26.7)	0.002^b^
Delirium risk-increasing drugs per patient [n]	0 (0–1)	2 (0–3)^c^	< 0.001^a^	0 (0–1)	1 (0–2)^d^	0.008^a^
Intake of delirium risk-increasing drugs [n]	98 (34.3)	33 (78.6)	< 0.001^b^	77 (41.0)	19 (63.3)	0.022^b^
Heavy alcohol use						
yes	28 (9.8)	7 (16.7)	0.178^b^	22 (11.7)	4 (13.3)	0.798^b^
no	258 (90.2)	35 (83.3)		166 (88.3)	26 (86.7)	
Smoking status						
yes	31 (10.8)	3 (7.1)	0.463^b^	16 (8.5)	2 (6.7)	0.733^b^
no	255 (89.2)	39 (92.9)		172 (91.5)	28 (93.3)	

Values are expressed as number (%) or median (interquartile range)

a Mann-Whitney U test comparing patients with and without POD

b Chi^2^-Test comparing patients with and without POD

c The following top 5 drug classes were observed: antidepressants (22.5%), opioids (14.6%), antiepileptic drugs (11.2%), antipsychotics (10.1%), and anti-Parkinson drugs (10.1%)

d The following top 5 drug classes were observed: antidepressants (19.0%), anti-Parkinson drugs (14.3%), opioids (11.9%), antiepileptic drugs (11.9%), and antipsychotics (11.9%)

POD = postoperative delirium; eGFR = estimated glomerular filtration rate; ACB = anticholinergic burden

### Development of a predictive risk score for POD including delirium risk-increasing drugs

Univariable logistic regression analysis was performed for all potential risk factors and the presence or absence of delirium. The continuous variables age, eGFR, ACB score, and number of delirium risk-increasing drugs were significantly associated with the development of POD (*p* < 0.001). The risk factors not significant and thus excluded from further calculations were sex (*p* = 0.465), heavy alcohol use (*p* = 0.183), and smoking status (*p* = 0.467).

Significant continuous factors were transformed into categorical variables based on suitable cut-off values determined through clinically established definitions or the Youden index of ROC analysis (number of delirium risk-increasing drugs = 1.5; age = 73.5 years; eGFR = 58.5 ml/min/1.73m^2^) rounded to a reasonable value. The final categorical variables were age 66–75 years (*p* = 0.02), age > 75 years (*p* < 0.001), eGFR < 60 ml/min/1.73m^2^ (*p* < 0.001), ACB score ≥ 3 (*p* < 0.001), and ≥ two delirium risk-increasing drugs (*p* < 0.001).

**Fig. 1:**
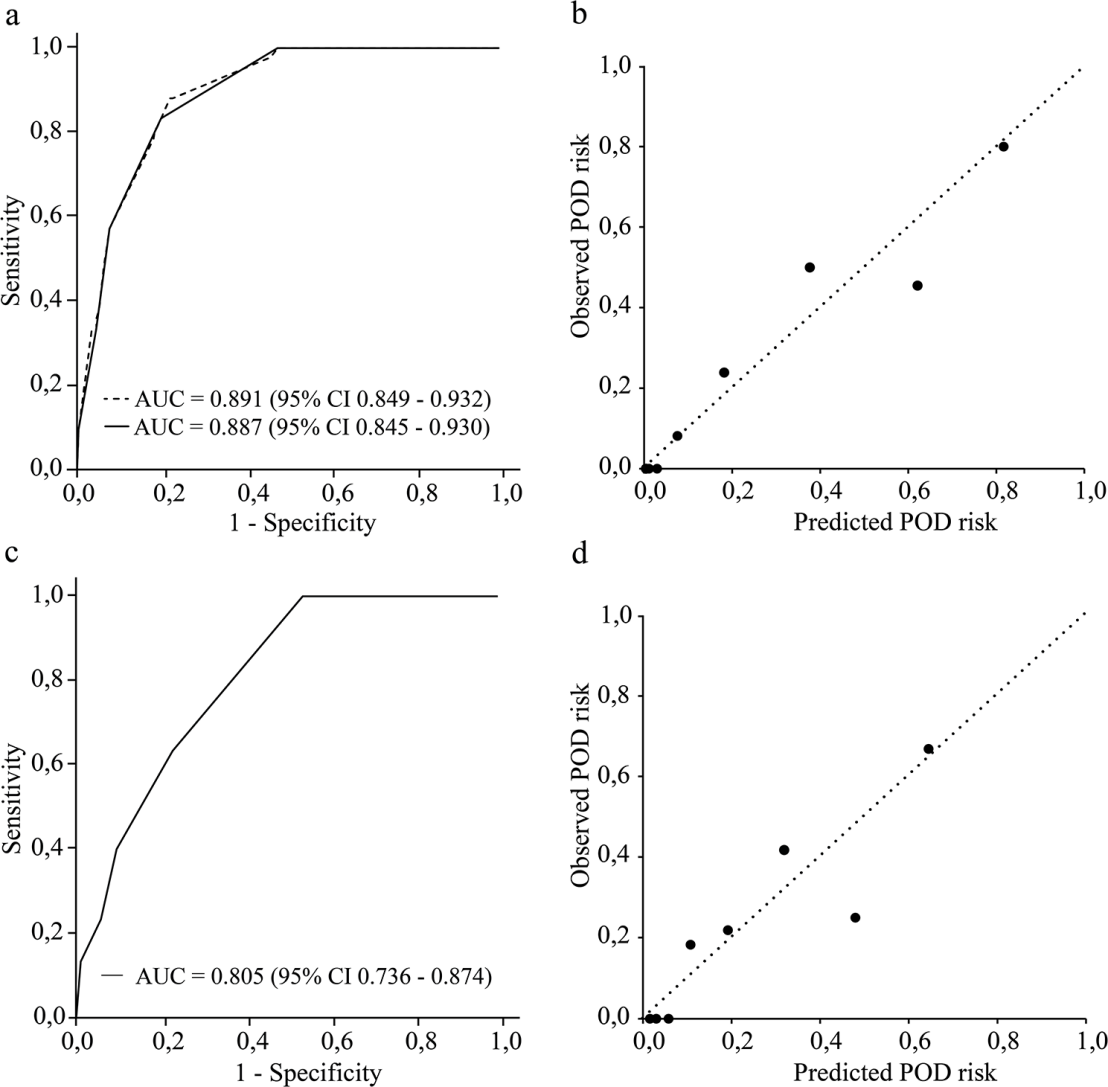
Receiver operating characteristic (ROC) curve, calculated area under the curve (AUC), and calibration plot for development and validation cohort. (**a**) Development cohort, (dotted line, ROC curve of the logistic regression model; solid line, ROC curve of the DRD score). (**b**) Development cohort, calibration plot comparing the predicted POD risk and observed POD risk. Patients were grouped into 8 groups of predicted risk according to the DRD score (0–7 points), the identity line is shown as dashed line. (**c**) Validation cohort, ROC curve of the DRD score. (**d**) Validation cohort, calibration plot AUC = area under the curve; ROC = receiver operating characteristic; CI = confidence interval; POD = postoperative delirium

These significant variables were further included in a multivariable forward stepwise logistic regression model. For derivation of the score, the corresponding regression coefficients were rounded to the nearest integer (Table 3). In this manuscript, this new score will be called the DRD score (risk score for POD including Delirium risk-increasing Drugs). Correlations between predictor variables were low (*r* < 0.8), indicating no multicollinearity [35]. The AUCs of the ROC curves of the logistic regression model and the derived DRD score are shown in Fig. 1a. The Hosmer-Lemeshow test indicated a good model fit (*p* = 0.602). Figure 1b shows the calibration plot comparing the predicted and observed POD risk. The optimal cut-off value for discriminating between patients at high and low risk for POD according to the Youden index was 3.5 points. Therefore, we classified patients at risk for drug-related POD who received ≥ 4 points.

**Table 3:** Independent risk factors for postoperative delirium identified by multivariable logistic regression analysis

Risk factor	Category	Regression coefficient	Odds Ratio (95% CI)	*p*	Points assigned
Age [years]	≤ 65		1		0
	66–75	2.41	11.15 (1.25–99.10)	0.03	2
	> 75	3.17	23.91 (2.80-204.26)	0.004	3
eGFR [ml/min/1.73m^2^]	≥ 60		1		0
	< 60	1.36	3.89 (1.21–12.11)	0.001	1
ACB score	< 3		1		0
	≥ 3	1.34	3.83 (1.21–12.11)	0.02	1
Delirium risk-increasing drugs [n]	< 2		1		0
	≥ 2	1.68	5.38 (2.28–12.65)	< 0.001	2

CI = confidence interval; eGFR = estimated glomerular filtration rate; ACB = anticholinergic burden

### Validation of the predictive risk score for POD including delirium risk-increasing drugs

To validate the developed risk score, we retrospectively applied the DRD score to each patient in the validation cohort. The AUC of the ROC curve of the validation cohort based on the risk score and the calibration plot are shown in Fig. 1c and d. There were no statistically significant differences between the patient characteristics of both cohorts in terms of the distribution of preoperative risk factors included in the developed score, as shown in Table 4. The sensitivity, specificity, PPV, NPV, and likelihood ratios for the DRD score were calculated for both the development and validation cohort and are shown in Table 5.

**Table 4:** Homogeneity between the development and validation cohorts for preoperative risk factors

Preoperative risk factor	Development cohort (*n* = 328)	Validation cohort (*n* = 218)	*p*
Age [years]			0.373
≤ 65	100 (30.5)	60 (27.5)	
66–75	84 (25.6)	49 (22.5)	
> 75	144 (43.9)	109 (50.0)	
eGFR [ml/min/1.73m^2^]			0.983
≥ 60	248 (75.6)	165 (75.7)	
< 60	80 (24.4)	53 (24.3)	
ACB score			0.763
< 3	296 (90.2)	195 (89.4)	
≥ 3	32 (9.8)	23 (10.6)	
Delirium risk-increasing drugs [n]			0.588
< 2	265 (80.8)	172 (78.9)	
≥ 2	63 (19.2)	46 (21.1)	

Values are expressed as number (%)

eGFR = estimated glomerular filtration rate; ACB = anticholinergic burden

**Table 5:** Performance of the predictive DRD score

	Development cohort (*n* = 328)		Validation cohort (*n* = 218)	
POD	Yes 42 (12.8)	No 286 (87.2)	Yes 30 (13.8)	No 188 (86.2)
High risk of POD (score ≥ 4)	35	57	19	43
Low risk of POD (score < 4)	7	229	11	145
Sensitivity (%)	83.3		63.3	
Specificity (%)	80.1		77.1	
Positive predictive value (%)	38.0		30.6	
Negative predictive value (%)	97.0		92.9	
Positive Likelihood ratio (LR+)	4.1		2.8	
Negative Likelihood ratio (LR-)	0.2		0.5	

Values are expressed as absolute number or number (%)

POD = postoperative delirium

## Discussion

We developed and validated a new predictive risk score for postoperative delirium including delirium risk-increasing drugs (DRD score) based on preoperative risk factors available during pharmacist-led medication reconciliation at hospital admission. In a retrospective single-centre study including orthopaedic and trauma surgery patients, the four risk factors advanced age, reduced kidney function, high anticholinergic burden, and number of delirium risk-increasing drugs proved to be predictive in the final model after multivariable logistic regression analysis. The sensitivity of the score was good with 83.3% in the development and fair with 63.3% in the validation cohort, also a good specificity was achieved with 80.1% and 77.1%, respectively. Thus, the newly developed DRD score is a promising tool for the early and pragmatic identification of patients at risk for POD. After calculation during medication reconciliation at admission it allows a timely initiation of preventive measures. Considering the severe clinical consequences of POD and that 40% of delirium cases are possibly preventable [30], implementation of the score in the clinical routine has the potential to considerably improve patient safety.

Drugs are a well-described risk factor for delirium. Psychoactive drugs and drugs associated with brain-related adverse effects are commonly known and possibly modifiable risk factors for delirium [11, 14, 36]. Surprisingly, in several predictive risk scores developed in recent years, drugs are mostly neglected [25, 32, 33, 37], although medications may account for 12–39% of delirium cases [38]. Risk scores including drugs either require complex automated calculations [16] or show an oversimplified approach when only considering polymedication (≥ five regular drugs) [13]. Our list of delirium risk-increasing drugs includes drugs with effects on the central nervous system as well as drugs correlating with comorbidities associated with delirium (i.e. diabetes mellitus and COPD) [14, 34, 39]. In our analysis, we found that taking two or more delirium risk-increasing drugs was a significant risk factor for POD (OR 5.38, 95% CI 2.28–12.65). Identifying these drugs during PhMR appears feasible and easily applicable in clinical practice.

Neurotransmitter disturbance is a major mechanism in delirium pathophysiology, and a reduced cholinergic activity is associated with altered attention and delirium [2]. Anticholinergic drugs that decrease central cholinergic activity can therefore increase the risk for delirium. This anticholinergic activity can be estimated through anticholinergic burden scales. A preoperative high anticholinergic burden is significantly associated with incident delirium [40–42], although contrary findings with no association have been reported in other studies [43]. Our study determined that a high anticholinergic burden was significantly associated with POD in both univariable and multivariable analyses.

Advanced age is a well-known risk factor for delirium [2, 7] and, accordingly, was proven to be a statistically significant factor in our study, as confirmed by multivariable analysis. However, thresholds for age as a risk factor vary in risk scores and evaluations published so far. In our study, two thresholds were evident: 66–75 years of age (OR 11.15) and > 75 years of age (OR 23.91); both of these thresholds were included in the score with distinct point assignments.

We found that a moderately decreased kidney function (eGFR < 60 ml/min/1.73m^2^) on the day of admission was significantly associated with POD (OR 3.89, 95% CI 1.21–12.11). To our knowledge, this predictor has not been considered in previously developed risk scores. An association between moderate renal impairment (eGFR 30–60 ml/min/1.73m^2^, calculated using cystatin-based equations) and delirium was found in fracture patients aged 75–84 years [44]. End-stage renal failure was a consistent risk factor for POD, as reported in an umbrella review of systematic reviews [45]. We used a creatinine-based CKD-EPI equation to calculate the eGFR and found that moderately decreased values were associated with delirium.

Although male gender is included in previously developed risk scores [33, 37, 46], we did not determine this factor to be significant. The predictors smoking and heavy alcohol use are also represented in published risk models [14, 25, 32, 33], whereas for our cohort no associations were found. Underreporting might be a reason for this finding since documentation was based on self-reports. Although the exact correlation between smoking and delirium is unclear, acute nicotine withdrawal may increase the risk of POD [47, 48]. Since this might be especially relevant to patients with a high nicotine dependency, binary reporting of the smoking status may be inadequate.

The new DRD score was developed and validated in a cohort of orthopaedic and trauma surgery patients who are known to be at risk for POD due to multiple risk factors [49, 50]. The prevalence of POD in our study was 18.4% for patients > 65 years, which is comparable to previous findings. The overall incidence of delirium in hospitalized older adults was 23% according to a meta-analysis of 33 studies [2]. The incidence of POD varies depending on the type of surgery with ≥ 20% for major surgery, which includes interventions in orthopaedic and trauma surgery [2]. Due to the retrospective assessment of POD through documented 4AT scores, ICD-10 diagnosis, and chart review, underreporting is possible due to inappropriate documentation, especially for patients with hypoactive delirium. Nonetheless, a chart-based method for identifying POD is validated and frequently used [51].

In this study, patients with a DRD score of 4 or higher were classified as at risk for POD. This applies for all patients > 65 years with an intake of at least two delirium risk-increasing drugs. For patients with less than two delirium risk-increasing drugs, depending on age, four points can only be reached if one or both additional risk factors (high anticholinergic burden and reduced kidney function) apply. For the predictive performance of the DRD score in the development and validation cohorts, sufficient AUC-values with 0.887 (95% CI 0.845–0.930) and 0.805 (95% CI 0.736–0.874) were obtained. Also calibration plots showed good calibration for both test cohorts (Fig. 1). The specificity and NPV were good in both development and validation cohort, meaning patients without risk will be stratified correctly. The sensitivity was good in the development cohort (83.3%), but lower in the validation cohort (63.3%), meaning some patients at risk could be missed. However, since the DRD score will be a first screening during PhMR and additional screening will take place on the ward, the achieved sensitivity was judged as acceptable. For patients identified at high risk for drug-related POD, pharmacists can consequently perform a medication review and state suggestions for drug therapy safety as an additional preventive measure to reduce the risk for POD.

Our study has several limitations. Since we performed a single centre study in a specific patient cohort of orthopaedic and trauma surgery patients, the generalizability of our findings is unknown and should be addressed in further studies. A number of important predictors for POD are not included in the DRD score, such as dementia, cognitive impairment, previously developed delirium, hearing and visual impairment, physical status, type of surgical procedure, and severity of illness [7, 25, 32, 33, 37, 46]. This is primarily due to its focus on implementation during PhMR, and we were thus limited to factors available at this time point. Although some patients with dementia receive anti-dementia drugs, we are aware that patients with unrecognized or untreated dementia will not be assessed. Patients who develop POD because of other, not drug-related risk factors might not be predicted through the DRD score. However, the focus of our new score includes delirium risk-increasing drugs and it could be a trigger for pharmaceutical advice with the aim to erase or minimize the risk for drug-related POD. Thus, the primary aim of the DRD score is to identify patients at risk for drug-related POD who may benefit from suggestions for drug therapy safety.

There is some overlap between our list of delirium risk-increasing drugs and drugs included in the ACB score. For receiving corresponding score points, at least two delirium risk-increasing drugs or an ACB score ≥ 3 are necessary. An ACB score ≥ 3 can either be reached for multiple drugs with a low to moderate anticholinergic effect or for single drugs with a high anticholinergic effect (e.g. tricyclic antidepressants or antimuscarinic agents for the treatment of overactive bladder). For drugs with high anticholinergic properties, which are also classified as delirium risk-increasing drugs, a double rating will only occur if other delirium risk-increasing drugs are taken in addition. Besides anticholinergic properties, delirium risk-increasing drugs have various other effects on the central nervous system or correlate with comorbidities associated with POD. Thus, cases with a double rating can be justified, and an additional risk for POD can be proposed due to multiple drug-related central nervous effects and associated comorbidities. Furthermore, for both risk factors no statistical multicollinearity was determined and the overlap was therefore not considered to be decisive.

We performed an internal validation in orthopaedic and trauma surgery patients. External validation in different surgical patient cohorts is necessary to estimate the score performance in other settings and determine the generalizability of the DRD score. However, as a strength, this study was performed with real-life data and considered the feasibility in clinical practice.

## Conclusion

The new DRD score is a predictive risk score assessable during pharmacist-led medication reconciliation at hospital admission and is suitable for identifying patients at risk for drug-related POD. In addition to general preventive measures, specific preventive measures concerning drug therapy safety should be implemented for identified patients to reduce the risk for POD.

### Electronic supplementary material

Below is the link to the electronic supplementary material.

Supplementary Material 1

## Data Availability

The data that support the findings of this study are available from the corresponding author upon reasonable request.

## Declarations

### Guidelines

The ‘Transparent reporting of a multivariable prediction model for individual prognosis or diagnosis’ (TRIPOD) reporting guideline [52] was followed during the study.
