# Supplementary material for: Development and validation of a new drug-focused predictive risk score for postoperative delirium in orthopaedic and trauma surgery patients
Source: BMC Geriatr. 2024 May 13;24:422. doi: 10.1186/s12877-024-05005-1 (PMC11092087; doi:10.1186/s12877-024-05005-1)
Supplement: Supplementary file 1 — Supplementary Material 1 [file 12877_2024_5005_MOESM1_ESM.pdf]

Table S1 Overview of observed drug classes of delirium risk-increasing drugs in both study cohorts and in total for patients with or without postoperative delirium

	**Development cohort n=346**		**Validation cohort n=218**		**Total n=546**	
Postoperative delirium	Yes	No	Yes	No	Yes	No
Patients (n)	42	286	30	188	72	474
Delirium risk-increasing drugs (n)	89	174	42	147	132	318
Antidepressants (n)	20 (22.5)	33 (19)	8 (19.5)	35 (23.8)	28 (21.4)	68 (21.2)
Opioids (n)	13 (14.6)	37 (21.3)	5 (11.9)	30 (20.4)	18 (13.7)	67 (20.9)
Antiepileptic drugs (n)	10 (11.2)	20 (11.5)	5 (11.9)	22 (15.0)	15 (11.5)	42 (13.1)
Antipsychotics (n)	9 (10.1)	13 (7.5)	5 (11.9)	13 (8.8)	14 (10.7)	26 (8.1)
Anti-Parkinson drugs (n)	9 (10.1)	6 (3.4)	6 (14.3)	6 (4.1)	15 (11.5)	12 (3.7)
Anti-dementia drugs (n)	8 (9)	0 (0)	1 (2.4)	2 (1.4)	9 (6.9)	2 (0.6)
Oral antidiabetics / GLP-1 analogues (n)	5 (5.6)	24 (13.8)	4 (9.5)	17 (11.6)	9 (6.9)	44 (13.7)
Hypnotics and sedatives (n)	4 (4.5)	14 (8)	2 (4.8)	9 (6.1)	6 (4.6)	23 (7.2)
Insulins and analogues (n)	4 (4.5)	9 (5.2)	1 (2.4)	4 (2.7)	5 (3.8)	13 (4)
Anxiolytics (benzodiazepines) (n)	3 (3.4)	6 (3.4)	0 (0)	2 (1.4)	3 (2.3)	8 (2.5)
Inhalants for chronic obstructive airway disease (COPD) (n)	3 (3.4)	7 (4)	3 (7.1)	3 (2.0)	6 (4.6)	10 (3.1)
Antihistamines for systemic use (n)	1 (1.1)	5 (2.9)	2 (4.8)	4 (2.7)	3 (2.3)	9 (2.8)

Values are expressed as number (%)
